# Bioinformatics analysis to identify potential biomarkers for the pulmonary artery hypertension associated with the basement membrane

**DOI:** 10.1515/biol-2022-0730

**Published:** 2023-09-26

**Authors:** Qian Li, Hu Zhang

**Affiliations:** Department of Cardiac Surgery, The First Affiliated Hospital of Kunming Medical University, Kunming 650000, China; Department of Cardiology, The First Affiliated Hospital of Kunming Medical University, Kunming 650000, China

**Keywords:** pulmonary arterial hypertension, basement membrane, immune infiltration analysis, bioinformatics analysis, ceRNA network

## Abstract

Pulmonary arterial hypertension (PAH) is a rapidly progressing cardiopulmonary disease. It is characterized by increased pulmonary artery pressure and vascular resistance. The most notable histopathological characteristic is vascular remodeling. The changes in the basement membrane (BM) are believed to be related to vascular remodeling. It is crucial to identify potential biomarkers associated with the BM in PAH, to guide its treatment. The microarray datasets GSE117261 and GSE113439 were downloaded from the Gene Expression Omnibus. Two data sets were examined to identify genes associated with the BM by analyzing gene expression changes. Next, we analyzed the relevant genes in the Kyoto Encyclopedia of Genes and Genomes using Gene Ontology and Disease Ontology annotationand conducted pathway enrichment analysis. We conducted a protein–protein interaction network analysis on the genes related to BMs and used the cell cytoHubba plug-in to identify the hub genes. Furthermore, we conducted an immune infiltration analysis and implemented a histogram model. Finally, we predicted and analyzed potential therapeutic drugs for PAH and set up a miRNA network of genetic markers. Six candidate genes related to BMs, namely Integrin Subunit Alpha V, Integrin Subunit Alpha 4, ITGA2, ITGA9, Thrombospondin 1, and Collagen Type IV Alpha 3 Chain, were identified as potential modulators of the immune process in PAH. Furthermore, ginsenoside Rh1 was found to significantly impact drug targeting based on its interactions with the six BM-related genes identified earlier. A novel biomarker related to the BM, which plays a crucial role in the development of PAH, has been identified.

## 1 Introduction

Pulmonary arterial hypertension (PAH) is a severe pulmonary vascular condition. It differs from hypertension. Hypertension is an increase in blood pressure resulting from increased arterial vascular resistance or decreased cardiac pumping activity. It is typically expressed as systolic blood pressure (diastolic blood pressure) ≥140 (90) mmHg, while PAH is caused by an increase in pulmonary vascular resistance or a decrease in pulmonary blood flow [1,2]. The primary histopathological characteristic of PAH is vascular remodeling. This leads to increased right ventricular afterload and may result in the disease rapidly advancing to heart failure [3,4]. Currently, the clinical treatment of PAH relies on drug therapy, including calcium antagonists, angiotensin-converting enzyme inhibitors, and prostaglandin drugs, among others [5,6]. These drugs primarily inhibit pulmonary vasoconstriction and control symptoms but have a limited impact on pulmonary vascular remodeling. Individuals with PAH typically have an unfavorable long-term outlook. Recent studies indicate that pulmonary vascular remodeling has a significant impact on PAH. Consequently, inhibiting or delaying the process has become a crucial strategy in the development of therapeutic drugs for the condition [7].

During the early stages of PAH formation, endothelial cells (ECs) can undergo damage and apoptosis. However, as the disease progresses, ECs seem to become resistant to apoptosis [8]. During the advanced stages of PAH, many ECs undergo excessive proliferation and accumulate anti-apoptotic properties. This accumulation eventually leads to the formation of plexiform lesions that can destroy the normal vascular structure [9]. The basement membrane (BM) is a special extracellular matrix (ECM), which is mainly composed of type IV collagen, laminin, proteoglycans (including BM heparin sulfate proteoglycans and other glycoproteins), and so on [10]. Laminin is the primary structural protein of the BM and is mainly responsible for the adhesion of epithelial cells to BM. Collagen type IV is a collagen that is specific to the BM and forms the structural scaffold of the BM. Proteoglycan is a complex long-chain glycoprotein with a high negative charge. This protein regulates the negative charge and biological properties of the BM and participates in signal transduction [10,11]. Therefore, BM ensures the integrity and stability of the vascular structure by preserving the normal positional relationship between the cells of the vascular wall and the ECM. Alterations in the BM have been observed in lung disorders that are distinguished by vascular remodeling and pulmonary hypertension [12]. Recent studies indicate a possible link between autoimmune diseases and autoantibody attacks against BM proteins. Furthermore, the expression and variation of BM have been found to significantly impact the development of PAH [13].

Bioinformatics research has been employed to investigate the possible pathogenetic factors for cardiovascular disease [14]. Pathogenic factors may include genetic susceptibility, with vascular lesions potentially caused by abnormalities in certain pathological genes and signaling pathways [15]. The mechanism of immune inflammation and the vascular inflammatory response is mediated by inflammatory factors and immune cells [16]. Vascular damage causes metabolic syndrome, obesity, insulin resistance, and other related metabolic disorders [17]. Currently, bioinformatics research in the PAH field is concentrated on mRNA expression profiling, exploring the molecular mechanisms underlying PAH, and identifying biomarkers through changes in gene expression in PAH patients. Ma et al. [18] found that 150 differently expressed genes (DEGs) of PAH were identified by Gene Expression Omnibus (GEO). It is speculated that Structural Maintenance of Chromosomes 2 (SMC2), DNA Topoisomerase II Alpha, SMC2, KIF23, and other targets may be involved in the pathogenesis of PAH, and the potential molecular mechanism of PAH provides new evidence. Nevertheless, there is currently no confirmed relationship between the BM genes and the pathogenesis of PAH. This is the first bioinformatics technique to identify the interaction between the hub genes of BM and lung tissue, which offers a novel viewpoint and reference for the management and treatment of PAH.

## 2 Materials and methods

### 2.1 Acquisition of genes associated with the BM through microarray data analysis

Gene expression data and microarray platform information for pulmonary tissues of PAH and normal controls (NC) were collected from the GEO database (https://www.ncbi.nlm.nih.gov/geo/) using the search term “Pulmonary Arterial Hypertension.” The criteria for data inclusion were: (a) the organism was of Homo sapiens origin, (b) all NC and pulmonary tissue samples had comprehensive data, and (c) the samples included both control and pulmonary tissues. We only analyzed datasets that met these criteria. Therefore, we included two datasets, GSE117261 and GSE113439, which exhibited similarities in their sequencing methods. A search of the literature resulted in the identification of 224 genes associated with BM for this experiment [19].

### 2.2 A screening of genes related to BM associated with PAH

The “sva” and “affy” R packages were used to standardize the downloaded sample data, which was then aggregated. After that, normalization and log2 transformation procedures were performed. We obtained gene expression data for BM from two distinct datasets and conducted a differential expression analysis using the “Limma” package. This investigation considered |log2FC| > 0.2 and adj. *P*.Val < 0.05 as statistically significant. The analysis of differential expression was categorized based on the sample type, which consisted of PAH and NC.

### 2.3 Analysis of gene enrichment related to the BM in PAH

We evaluated the biofunctionality of Gene Ontology (GO), the occurrence of Disease Ontology (DO) disease, and the enrichment of Kyoto Encyclopedia of Genes and Genomes (KEGG) pathways. The “clusterProfiler” package in the R program was used to visualize the DEGs. The significance threshold was set at an adjusted *P* value of less than 0.05.

### 2.4 Construction of protein–protein interaction (PPI) network and identification of hub genes

The STRING tool was used to explore the PPI network of BM-associated genes in PAH [20]. Moreover, the interactions between biological molecules associated with PAH were visualized and analyzed via the Cytoscape software [21]. CytoHubba plug-in in Cytoscape software was used to calculate the number of nodes and edges in the PPI network and obtain the maximum clique centrality (MCC) value, Degree value, and other parameters of each node. In this study, the top 10 genes with the highest Degree were selected as hub genes.

### 2.5 Immunoassay of BM-related genes

The presence of 16 different immune cell types and the activity of 13 immune-related pathways in every patient with PAH were examined using the single sample gene set enrichment analysis (ssGSEA) method provided by the “gsva” R package [22]. Initially, we collected gene sets for 16 immune cells, such as T cells, B cells, natural killer cells, and macrophages, from the ImmPort database. In addition, we collected gene sets for 13 immune-related pathways, such as the TNF-α signaling pathway and the T-cell receptor signaling pathway, from the KEGG database. Subsequently, we quantified the level of immune infiltration of 16 immune cells in each sample using the ssGSEA method. Similarly, we quantified the enrichment scores for the 13 immune-related functional pathways in each sample using the ssGSEA method. Correlation analysis was performed using the Spearman correlation analysis; *P* < 0.05 represents a significant correlation.

### 2.6 Nomogram design and validation

To assess the impact of the hub gene on survival in patients with PAH, we constructed a nomogram model. The nomogram was generated using the R package “rms” and the R package “ROCR” was used to evaluate the predictive power of the model. These packages considered risk scores and the expression of genes linked to the BM.

### 2.7 Prediction of drugs and miRNAs for genes associated with the BM

The Enrichr database [23] (https://maayanlabcloud/Enrichr/) was used to screen for genes related to pulmonary hypertension and BM based on a pharmacological prediction threshold of *P* < 0.05. To investigate miRNA–mRNA associations within the competing endogenous RNA (ceRNA) network further, we used the Enrichr database to identify possible miRNAs that could target the Hub genes. Next, a network diagram of the ceRNA system was constructed using Cytoscape software.

## 3 Results

### 3.1 Analysis of genes exhibiting differential expression in PAH

**Figure 1:**
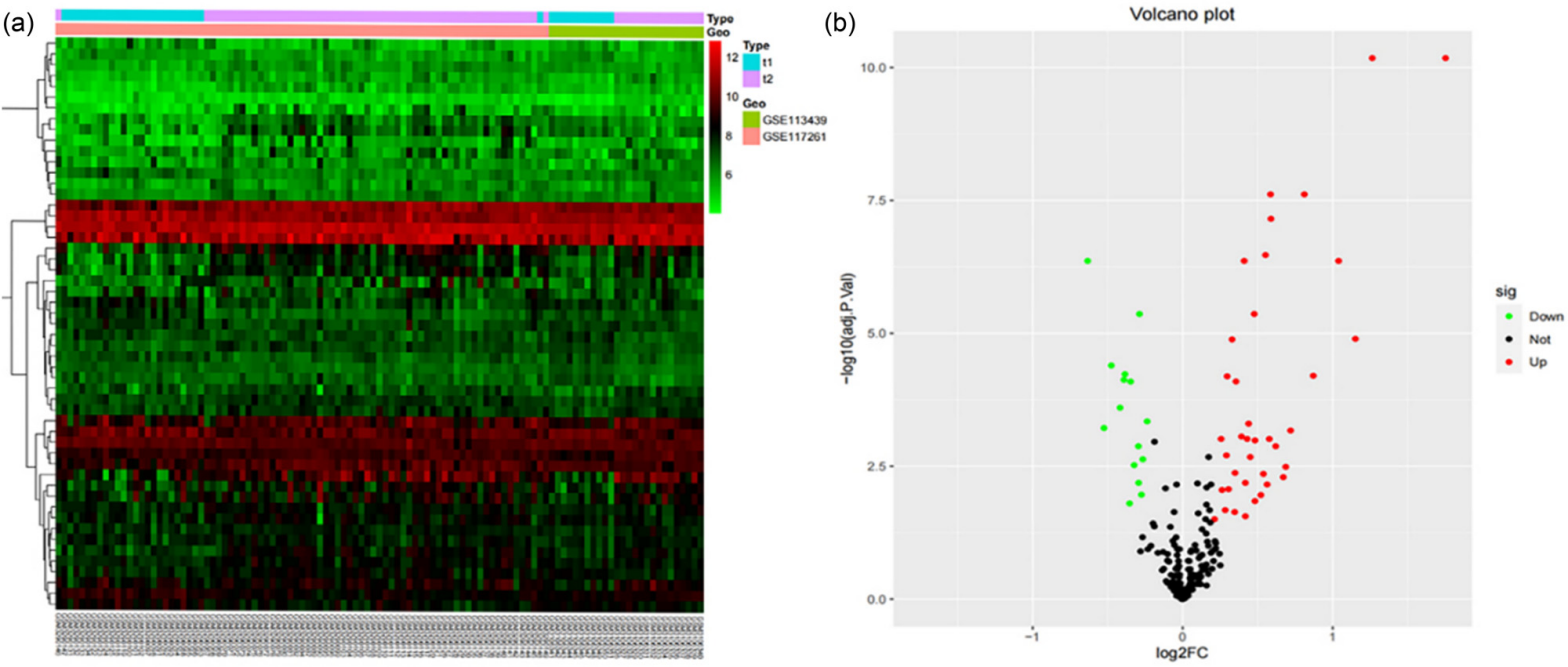
An analysis of DEGs in the BM between PAH and control samples was conducted. (a) Each row of the heat map represents one DEG, while each column represents a sample, which is either normal or PAH. The color red denotes upregulated DEGs and green denotes downregulated DEGs. (b) The red points represent upregulated genes, while the green points represent downregulated genes. Abbreviations: DEG, differentially expressed gene; PAH, pulmonary artery hypertension.

We downloaded the original expression profiles for patients with PAH for GSE117261 and GSE113439 from the GEO database. The GSE11726 dataset comprised 58 samples of PAH and 25 samples of normal lung (NC) tissue. The GSE113439 dataset contained 15 samples of PAH and 11 samples of NC tissue. We merged 36 NC samples and 73 PAH samples from two datasets, GSE117261 and GSE113439. In addition, we compared the genes related to BM with those related to PAH and conducted further analysis using criteria of |log2FC| > 0.2 and adj. *P*.Val < 0.05. Following this, the differential analysis showed that 53 DEGs, of which 36 upregulated genes and 17 downregulated genes, were differentially expressed between PAH samples and NC samples (refer to Figure 1a and b).

### 3.2 BM-related gene enrichment analysis

**Figure 2:**
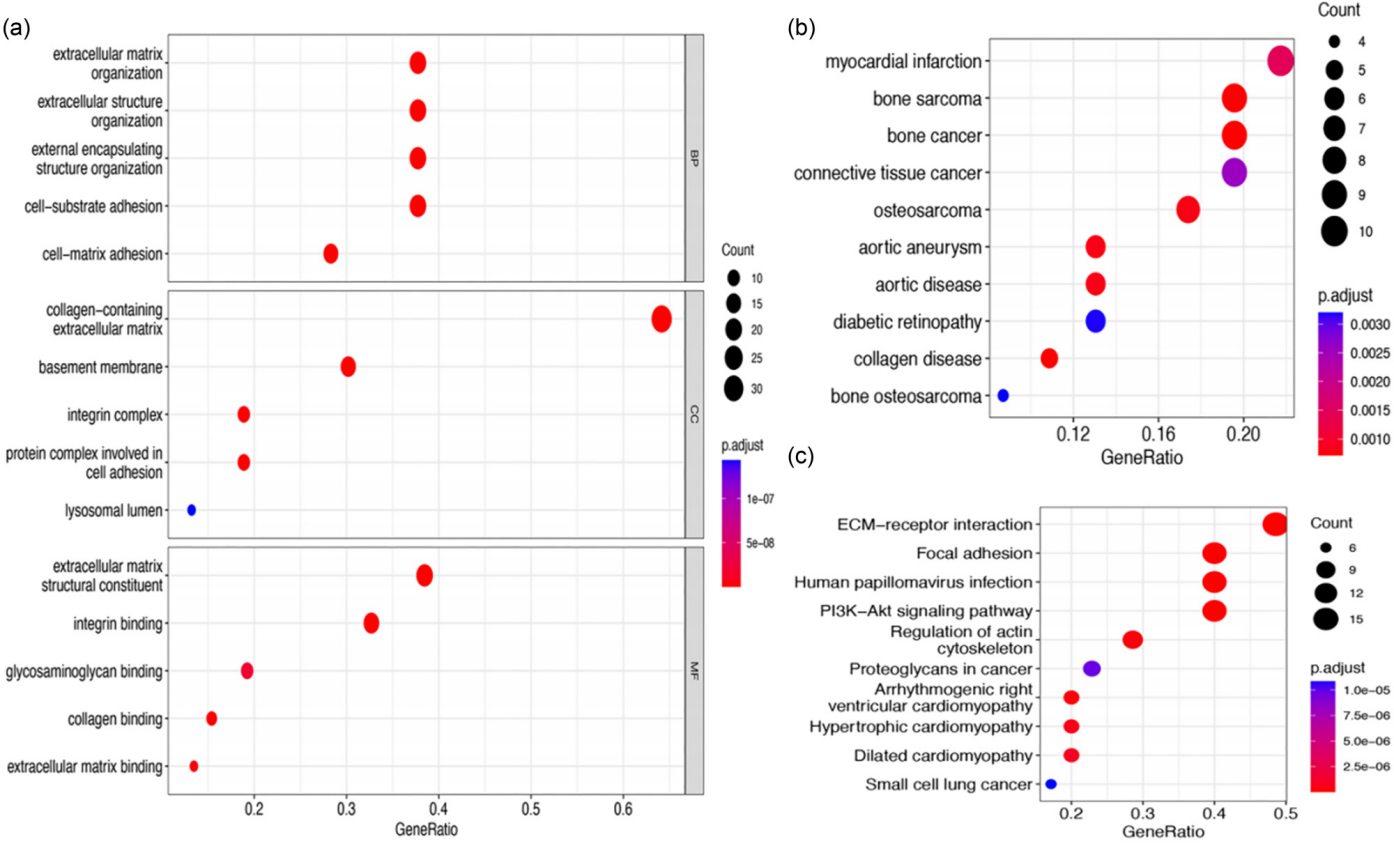
Enrichment analysis graph. (a) GO functional enrichment analysis; (b) DO disease enrichment analysis; and (c) KEGG pathway enrichment analysis.

Functional annotation of differential genes was performed, as shown in Figure 2. From the GO functional enrichment analysis, it was found that the DEGs involved in biological processes mainly included ECM organization, extracellular structure organization, external encapsulating structure organization, cell–substrate adhesion, and cell–matrix adhesion; the molecular components included collagen-containing ECM, BM, integrin complex, protein complex involved in cell adhesion, and lysosomal lumen; and the molecular functions included ECM structural constituent, integrin binding, glycosaminoglycan binding, collagen binding, and ECM binding (as shown in Figure 2a). BM-DEGs were found to be significantly enriched in myocardial infarction, bone sarcoma, bone cancer, and connective tissue cancer in DO enrichment analysis (Figure 2b). According to the results of KEGG enrichment analysis, BM-DEGs were mainly enriched in ECM–receptor interaction, focal adhesion, human papillomavirus infection, phosphoinositide 3-kinase (PI3K)-Akt signaling pathway, and the regulation of actin cytoskeleton (as shown in Figure 2c). Further information regarding the enrichment analysis can be found in Table 1.

**Table 1:** Enrichment analysis notes

Category	ID	Description	*P* value
BP	GO:0030198	ECM organization	2.22 × 10^−22^
BP	GO:0043062	Extracellular structure organization	2.36 × 10^−22^
BP	GO:0045229	External encapsulating structure organization	2.68 × 10^−22^
BP	GO:0031589	Cell–substrate adhesion	4.35 × 10^−21^
BP	GO:0007160	Cell–matrix adhesion	8.31 × 10^−17^
CC	GO:0062023	Collagen-containing ECM	7.08 × 10^−44^
CC	GO:0005604	BM	5.17 × 10^−25^
CC	GO:0008305	Integrin complex	3.15 × 10^−19^
CC	GO:0098636	Protein complex involved in cell adhesion	1.08 × 10^−16^
CC	GO:0043202	Lysosomal lumen	7.32 × 10^−09^
MF	GO:0005201	ECM structural constituent	8.87 × 10^−28^
MF	GO:0005178	Integrin binding	4.09 × 10^−23^
MF	GO:0005518	Collagen binding	1.68 × 10^−11^
MF	GO:0050840	ECM binding	1.95 × 10^−10^
MF	GO:0005539	Glycosaminoglycan binding	1.01 × 10^−09^
DO	DOID:0080639	Bone sarcoma	2.78 × 10^−06^
DO	DOID:184	Bone cancer	3.94 × 10^−06^
DO	DOID:854	Collagen disease	4.98 × 10^−06^
DO	DOID:3347	Osteosarcoma	7.49 × 10^−06^
DO	DOID:3627	Aortic aneurysm	9.93 × 10^−06^
DO	DOID:520	Aortic disease	1.15 × 10^−05^
DO	DOID:5844	Myocardial infarction	2.38 × 10^−05^
DO	DOID:201	Connective tissue cancer	4.77 × 10^−05^
DO	DOID:8947	Diabetic retinopathy	6.64 × 10^−05^
DO	DOID:3376	Bone osteosarcoma	7.43 × 10^−05^
KEGG	hsa04512	ECM–receptor interaction	2.44 × 10^−25^
KEGG	hsa04510	Focal adhesion	2.57 × 10^−14^
KEGG	hsa05165	Human papillomavirus infection	2.42 × 10^−11^
KEGG	hsa04151	PI3K–Akt signaling pathway	5.97 × 10^−11^
KEGG	hsa04810	Regulation of actin cytoskeleton	2.31 × 10^−08^
KEGG	hsa05412	Arrhythmogenic right ventricular cardiomyopathy	2.62 × 10^−08^
KEGG	hsa05410	Hypertrophic cardiomyopathy	7.81 × 10^−08^
KEGG	hsa05414	Dilated cardiomyopathy	1.22 × 10^−07^
KEGG	hsa05205	Proteoglycans in cancer	1.72 × 10^−06^
KEGG	hsa05222	Small cell lung cancer	2.09 × 10^−06^

Abbreviations: GO, Gene Ontology; BP, biological process; CC: cellular component; MF: molecular function; DO, Disease Ontology; KEGG, Kyoto Encyclopedia of Genes and Genomes.

### 3.3 PPI networks

**Figure 3:**
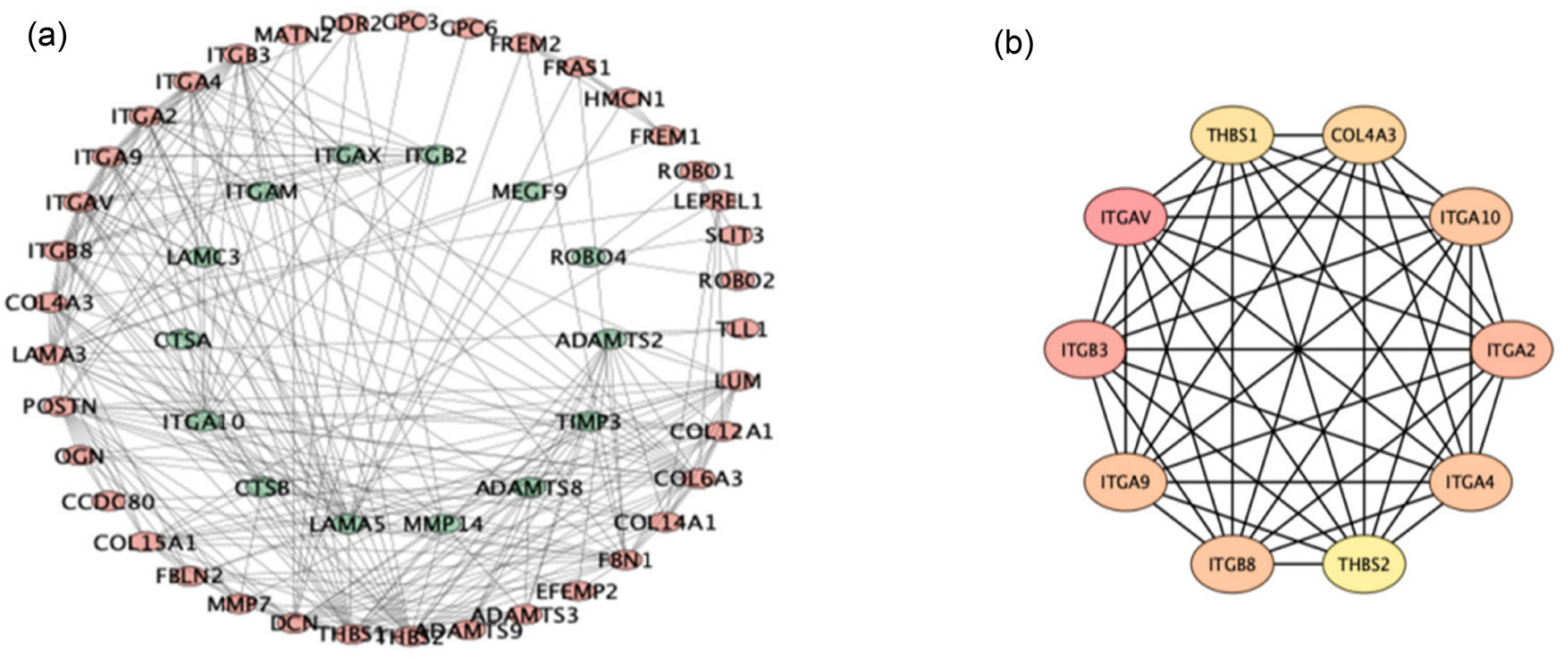
PPI network construction and hub gene screening. (a) The PPI network of DEGs was depicted using the Cytoscape software. Upregulated genes were labeled in red, and downregulated genes were labeled in green. (b) Ten hub genes were screened using the cytoHubba plug-in. Abbreviations: DEGs, differently expressed genes; PPI: protein–protein interaction.

To develop a deeper understanding of the correlation between BM-DEGs, a PPI network analysis was carried out. Figure 3 shows the resulting network consisting of 53 nodes, 272 edges, and an average node degree of 10.3. We used the cytoHubba algorithm to identify 10 hub genes: These hub genes are Integrin Subunit Alpha V (ITGAV), ITGB3, ITGA9, ITGB8, THBS2, Integrin Subunit Alpha 4 (ITGA4), ITGA2, ITGA10, Collagen Type IV Alpha 3 Chain (COL4A3), and Thrombospondin 1 (THBS1). All of the 10 genes mentioned above have MCC and degree values that are higher than the average value of all nodes in the network diagram.

### 3.4 Analysis of the immune cells and immune responses related to genes associated with the BM

**Figure 4:**
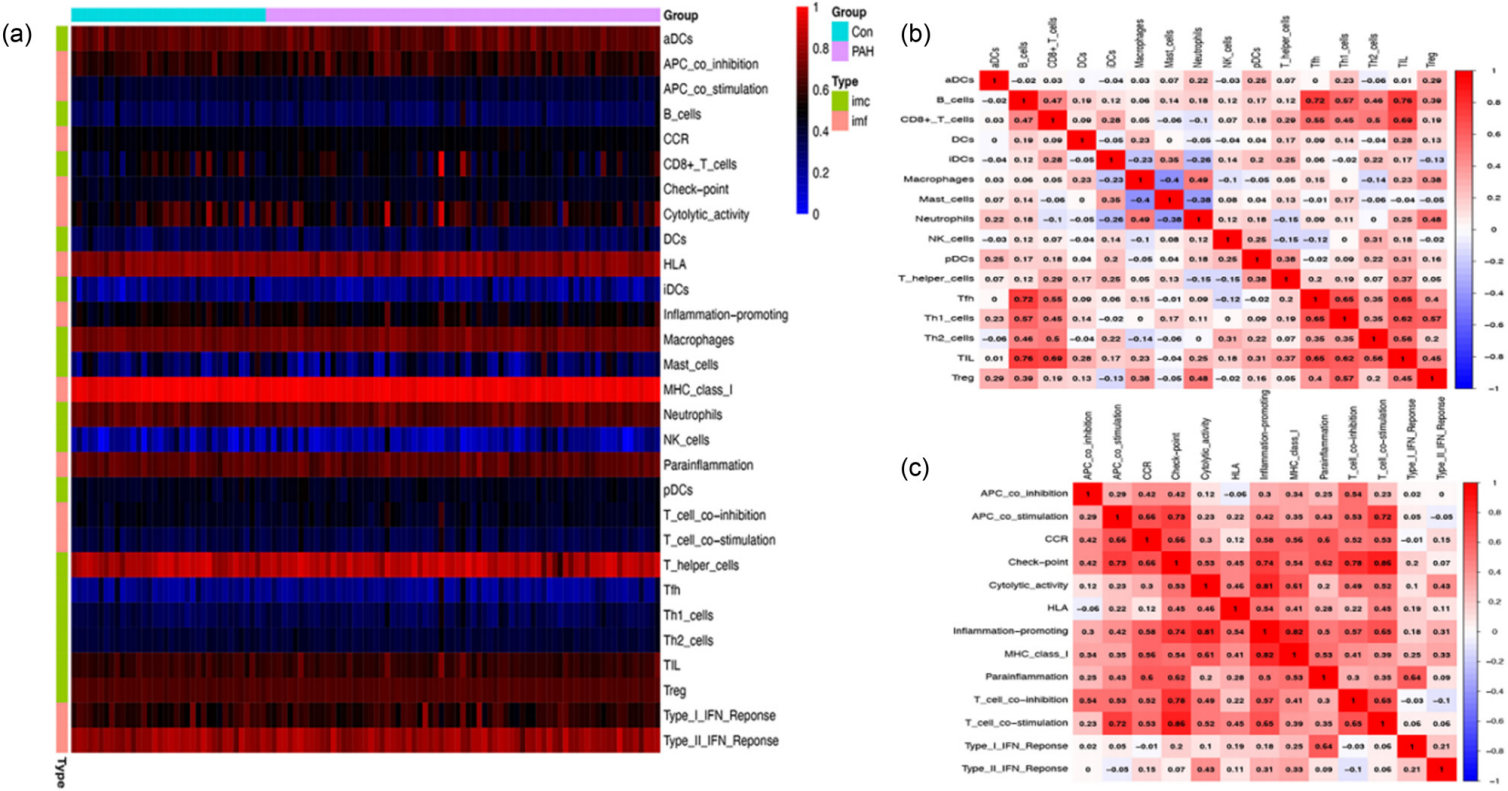
Immune cell infiltration analysis in PAH. (a) Visualization of the distribution of immune cells and their functional roles in PAH using a heat map. (b) Immune cell correlation heat map. (b and c) Immune-related function heat map.

**Figure 5:**
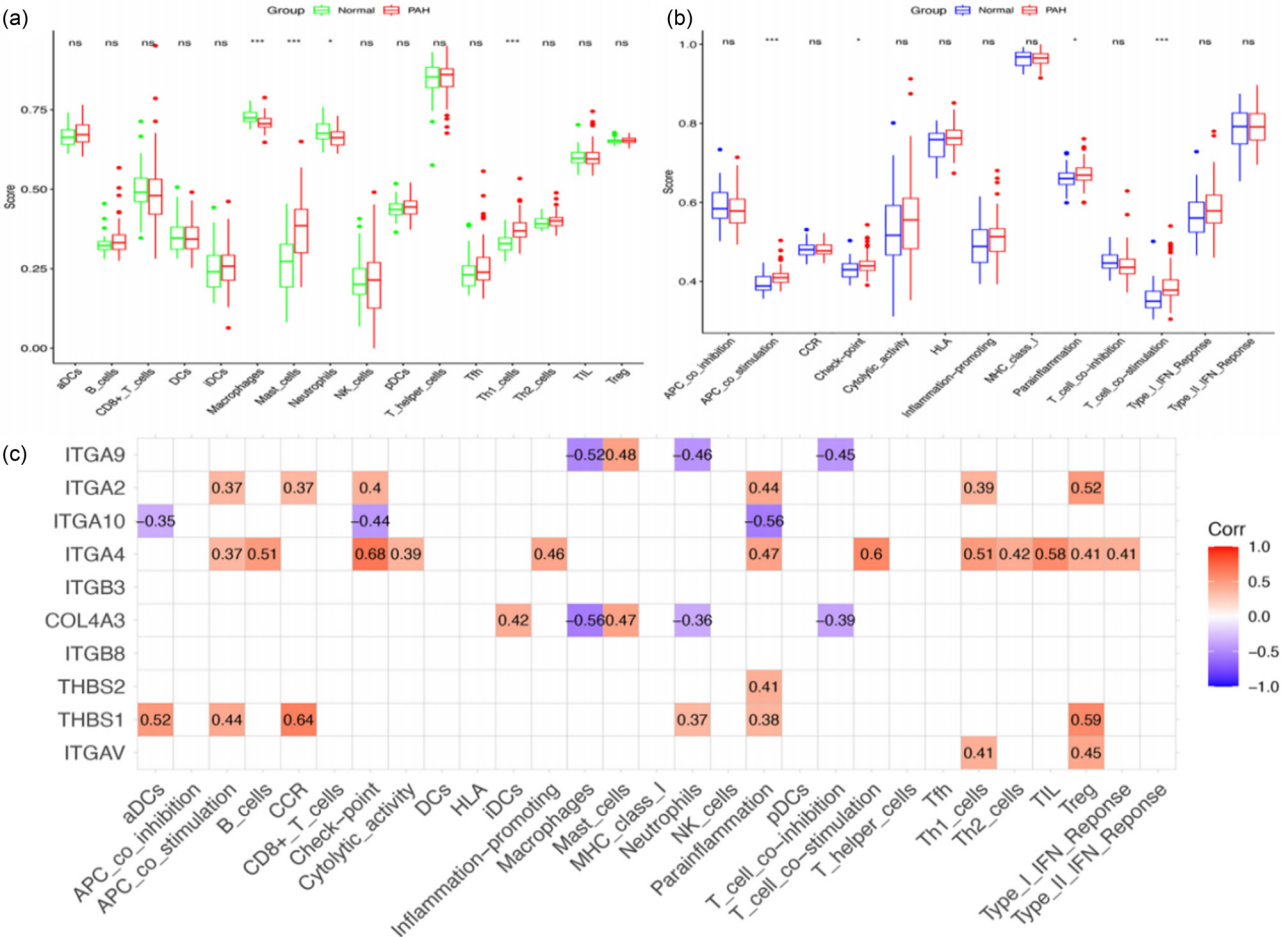
Distribution and visualization of immune cell infiltration. (a) Line plot depicting infiltration of immune cells. (b) Line plot showing immune-related function. (c) The correlation plot displays the correlation between the BM-related genes and immune cells as well as immune function (***P < 0.001, **P < 0.01, and *P < 0.5).

To investigate the immune status variations of hub genes in PAH, we divided the PAH dataset into two groups: NC and PAH. Afterwards, we evaluated the enrichment scores of immune cells and immune-related functional pathways for both groups. Figure 4a and b shows a positive correlation between B cells and both tumor-infiltrating lymphocytes (TIL) (*r* = 0.76) and follicular helper T cells (Tfh) (*r* = 0.72). In contrast, mast cells exhibited a negative correlation with both macrophages (*r* = 0.40) and neutrophils (*r* = 0.38). Figure 4c shows that immune-related functions positively correlate with both checkpoint and T-cell costimulation (*r* = 0.86). Additionally, inflammation-promoting factors were positively associated with major histocompatibility complex I (*r* = 0.82). In contrast, there was a negative correlation between T-cell co-inhibition and the type-II interferon response (*r* = −0.10), and APC-costimulation was negatively correlated with human leukocyte antigen (*r* = −0.06) as shown in Figure 4c. The levels of macrophages, mast cells, neutrophils, Th1 cells, and Tfh were significantly higher in the PAH group than in the NC group, as depicted in Figure 5a. Moreover, the levels of APC-costimulation, checkpoint, para-inflammation, and T-cell stimulation expression were significantly higher in the PAH group than in the NC group, as illustrated in Figure 5b.

To better understand the relationship between hub genes and the infiltration of immune cells in PAH, we used Spearman to analyze the correlation between hub genes and immune cell infiltration levels. Figure 5c shows a positive correlation between ITGA9 and mast cells (*r* = 0.48), and a negative correlation with macrophages, neutrophils, and T-cell co-inhibition (*r* = −0.52, −0.46, −0.45). ITGA2 showed a positive correlation with APC-costimulation, chemokine receptor (CCR), checkpoint, para-inflammation, Th1 cells, and Treg immune cells. There was an inverse relationship between para-inflammation (*r* = −0.56) and checkpoint (*r* = −0.44) with ITGA10. ITGA4 had positive correlations with checkpoint (*r* = 0.68), T-cell costimulation (*r* = 0.60), and TIL (*r* = 0.58). COL4A3 is positively associated with the immune cell type mast cells (*r* = 0.47) and was found to have a negative correlation with macrophages (*r* = −0.56). There was a positive correlation (*r* = 0.41) between THBS2 and immunological function para-inflammation. There was a positive correlation (*r* = 0.64) between immune cell CCR and THBS1, but THBS1 had a negative correlation (*r* = −0.59) with Treg. There was a positive relationship found between ITGAV and the immune cell types of Treg (*r* = 0.45) and Th1 cells (*r* = 0.41). Based on the results mentioned above, it is suggested that the hub gene plays a crucial role in regulating immune cell activity and responses in the immune system.

### 3.5 Construction and validation of nomogram

**Figure 6:**
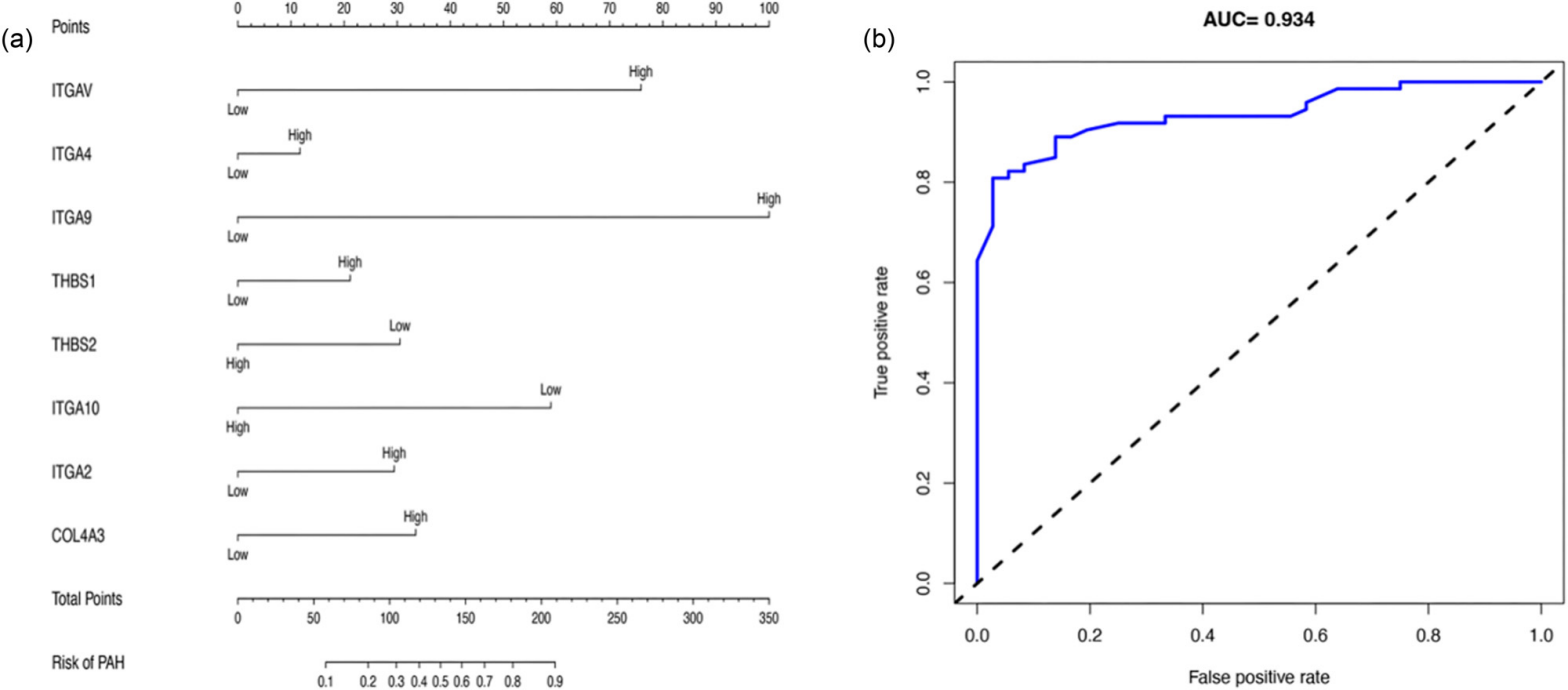
A nomogram is presented to predict the relationship between expression of hub genes and survival rate among individuals with PAH. (a) Survival nomogram prediction model for PAH patients (b) ROC curve to evaluate the accuracy of PAH prediction by a nomogram model.

To assess the effect of hub gene on PAH patients, we established a nomogram to predict the association between hub gene expression and survival in PAH patients. The model included the expression patterns of six BM-DEGs namely ITGAV, ITGA4, ITGA9, THBS1, ITGA2, and COL4A3. It also made use of the expression levels of six hub genes as variables, as illustrated in Figure 6a. The findings indicate that the nomogram predicted the area under the curve (AUC) value for the receiver operating characteristic curve (ROC) for PAH patients to be 0.934 (Figure 6b).

### 3.6 Prediction of drug and miRNA targets for genes associated with the BM

**Figure 7:**
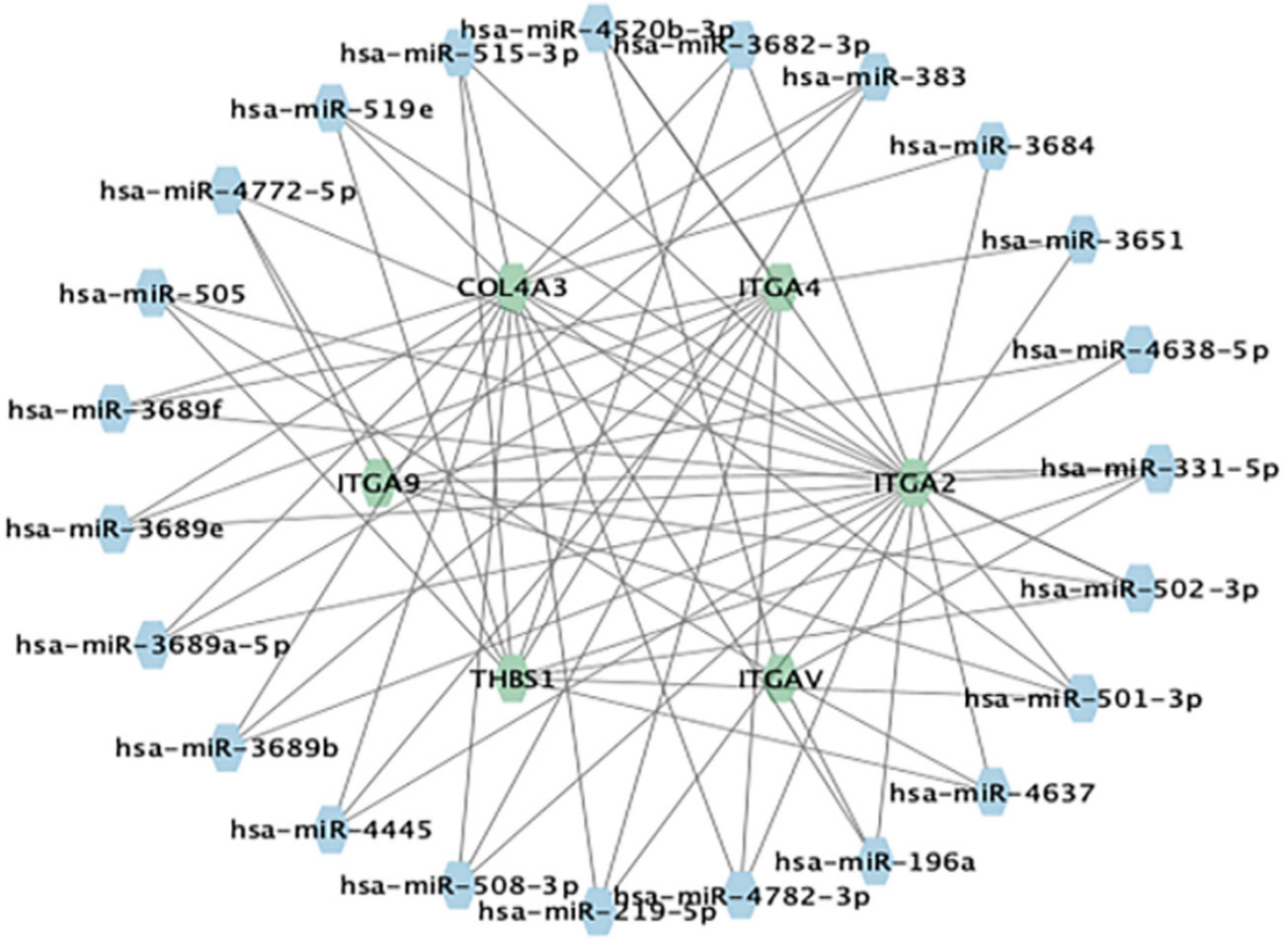
mRNA–miRNA regulatory network. A total of 25 miRNAs were anticipated to be linked with the hub genes.

We investigated possible therapeutic drugs for PAH that modify the hub gene by utilizing the Drug Signatures Database (DSigDB, http://tanlab.ucdenver.edu/DSigDB). A total of 361 target medications were identified. Table 2 displays the top 10 target medications, ranked by their composite score. To achieve a better understanding of the molecular basis of the six hub genes, we used the Enrichr database to predict their associated miRNAs. These hub genes were found to be associated with a total of 25 miRNAs. We used Cytoscape software, as shown in Figure 7, to build miRNA–mRNA networks.

**Table 2:** Drug prediction results of BM-associated genes

Term	Adjust *P*-value	Odds ratio	Combined score
Clopidogrelum [Latin] CTD 00002343	0.003400	453.91	4896.92
58-64-0 CTD 00005321	0.003400	384.00	4022.15
Sulfinpyrazone TTD 00011126	0.04229	399.68	2284.21
Ginsenoside Rh1 CTD 00003920	0.04229	399.68	2284.21
Cortisolsuccinate CTD 00000336	0.04229	399.68	2284.21
Epinephrine CTD 00005907	0.007588	216.83	2034.63
Tesmilifene CTD 00001953	0.04229	333.03	1847.77
Phenylbutazone TTD 00010195	0.04229	307.40	1682.80
Raltitrexed CTD 00002721	0.04229	285.43	1542.87
8-iso Prostaglandin A2 CTD 00003317	0.04229	235.02	1227.64

## 4 Discussion

Generally, the primary function of healthy pulmonary vessels is to uphold pulmonary circulation and facilitate lung gas exchange. Normal pulmonary vessels consist of ECs, intima, media, and adventitia structurally [8]. Vasodilation and smooth muscle relaxation are maintained by ECs through the production of nitric oxide, prostacyclin, and other factors [24]. The intima consists of smooth muscle cells that are sparsely arranged and can contribute to the regulation of vasoconstriction [25]. Elastic fibers make up the main component of the media layer, providing resistance to the distension and deformation of blood vessels. Fibroblasts and collagen are found in the adventitia layer, providing vascular support in terms of structure [26,27]. During the development of PAH, the normal vascular structure and function are gradually destroyed. PAH can be subdivided into three stages based on the progression of the disease: the compensatory stage, the decomposition stage, and the end stage [4]. During the compensatory phase of PAH, the pulmonary artery maintains vasodilation and low vascular tension. At the decompensation stage, pulmonary artery contraction worsened, and vascular reactivity decreased. Endothelial function became impaired, leading to a downregulation in the expression of nitric oxide synthase (eNOS). The proliferation of smooth muscle cells was evident. There is end-stage, severe, and irreversible pulmonary vasoconstriction and occlusion. The endothelium suffered severe damage, leading to a drastic reduction in eNOS expression and loss of the ability to synthesize prostacyclin. The proliferation of smooth muscle cells results in vascular stenosis. The blood vessels have lost almost all their reactivity [28]. Individual pulmonary vascular remodeling and changes in the BM are closely related to the development of PAH. Hence, it is crucial to determine the functions of DEGs in the BM of PAH patients to assess the severity of disease progression and suggest suitable treatment options.

This study examines two tissue microarray datasets (GSE117261 and GSE113439) of PAH from the GEO database, in conjunction with the analysis of BM-related genes, revealing DEGs between the PAH and NC groups. By constructing a PPI network, we identified the top 10 hub genes, including ITGAV, ITGB3, ITGA9, ITGB8, THBS2, ITGA4, ITGA2, ITGA10, COL4A3, and THBS1. These genes were mainly enriched in ECM organization, extracellular structure organization, collagen-containing ECM, BM, and ECM structural constituent. The ECM is a complex network of structural proteins like collagen and functional proteins like adhesion factors covering the cellular microenvironment. It actively participates in maintaining normal tissue structure and cell function [29]. ECM organization is the process of organizing the ECM, which includes synthesizing, depositing, and cross-linking matrix components. These processes collectively create a 3D microenvironment structure that promotes cell growth, migration, and function [30]. The study identified ECM organization as the most significant enrichment pathway, indicating that ECM remodeling may have a crucial role in this pathological process. Alterations in the expression and deposition patterns of matrix constituents may impact cell viability and tissue structure. The BM-DEGs were primarily enriched in myocardial infarction and osteosarcoma in DO. The KEGG enrichment analysis showed that BM-DEGs in PAH patients were mainly involved in ECM–receptor interaction, adhesion, human papillomavirus infection, the PI3K–Akt signaling pathway, and regulation of the actin cytoskeleton.

To better comprehend the role of immune cell infiltration in the development of PAH, we examined the correlation between immune cells, immune function, and DEGs in PAH with a focus on hub genes. The findings indicated that the hub gene could play an indirect role in the infiltration of immune cells in PAH by affecting immune cell CCR, T-cell costimulation, immune regulation pathways, checkpoints, and para-inflammation either directly or indirectly. This implies that the hub gene could influence the development of PAH. ITGAV located in the hub gene is expressed widely on the surface of connective tissue and immune cells. It may combine with various β-integrins to create a heterodimeric integrin that binds to ECM proteins, including fibronectin, osteocalcin, and fibronectin and contributes to cellular adhesion and migration [31]. Research has indicated that among patients with low-grade glioma, ITGAV mRNA expression levels are linked to tumor immunity, immune checkpoints, immune checkpoint blockade, and response to chemotherapy. An elevated expression of ITGAV is indicative of severe infiltration of inflammatory cells [32]. Integrin Alpha 4, also known as ITGA4, mediates the adhesion of lung tissue and the chemotactic migration of immune cells, including lymphocytes, monocytes, and neutrophils. Studies have found that ITGA4 is a crucial regulatory gene in the pathological process of systemic sclerosis-associated interstitial lung disease, which is associated with lung function [33]. A nomogram was created to forecast the correlation between hub genes and the incidence of PAH. The result’s accuracy improved with an AUC value of 0.934 for the ROC curve. An extensive network of interactions was established between miRNAs and hub genes to obtain a list of miRNAs and mRNAs. This process involved utilizing the Enrichr database to evaluate the six genes, leading to the identification of 25 miRNAs and 69 mRNA–miRNA pairs.

Among the six hub genes, ITGAV, ITGA4, ITGA9, and ITGA2 belong to the Integrin Alpha chain family. THBS1 is a glycoprotein gene, and COL4A3 is a gene for the Collagen Alpha 3 (IV) chain [34]. Integrin is a transmembrane receptor that plays a critical role in cell adhesion. Integrins connect the ECM with the cytoskeleton, facilitating the transmission of biochemical and mechanical signals between the cell and its surrounding environment [34]. In the development of pulmonary vascular remodeling leading to PAH, the following processes occur: various stimulating factors cause damage to pulmonary arterial vessels resulting in EC dysfunction and apoptosis during the early stages of PAH. As a result, vascular endothelial permeability increases, there is a massive aggregation of platelets and inflammatory factors, and pulmonary artery smooth muscle cells are activated. Smooth muscle cells proliferate and migrate uncontrollably, depositing in the intima and media of the blood vessels leading to thickening and hardening of the blood vessel wall. Simultaneously, damaged ECs release increasing amounts of pro-fibrotic factors. This causes abnormal proliferation of cells similar to myofibroblasts and excessive secretion of ECM. This resulted in a broken BM, which led to a large accumulation of collagen. Subsequently, the vascular wall became increasingly fibrotic. This gradual process leads to alterations in the structure and function of the pulmonary artery wall, further narrowing of the vascular lumen, and related hemodynamic changes. Subsequently, there is increased resistance of the pulmonary vascular system, which ultimately leads to the development of PAH [1,35]. The most prominent pathological characteristic is the significant hyperplasia of fibrous tissue in the intima of the pulmonary artery wall. Research on human patient samples and animal model studies has confirmed that numerous ECM components including, but not limited to, collagen, elastin, tenascin-c, and fibronectin are deposited in these hyperplastic fibrous tissues [36,37]. PAH caused by hypoxia is usually the major contributor to cardiovascular disease. The activation of the transforming growth factor-beta (TGF-β) signaling pathway is crucial for the development of hypoxic and other forms of PAH [38]. Studies conducted recently revealed that stability of HIF2α results in increased expression of a protein, thrombospondin-1 (TSP-1), which activates TGF-β [39]. Collagen type IV is a crucial constituent of the ECM including COL4A3 as one of its components [40]. To summarize, several genes related to the BM mentioned above possibly contribute to the development of PAH.

The abnormal growth and movement of endothelial and smooth muscle cells in the lungs of PAH patients is a significant pathological feature in the occurrence and development of PAH. Targeting the growth and movement of these cells has been considered an effective method to combat this situation and treat PAH. The published literature highlights the importance of the PI3K–Akt signaling pathway in the pathological progression of PAH [41]. The PI3K/Akt pathway’s role in promoting cell proliferation and survival has been extensively researched in various types of cells, including smooth muscle cells with abnormal proliferation [42]. Moreover, the possibility of treating PAH by targeting the mammalian target of rapamycin (mTOR) has been investigated. mTOR is a crucial downstream entity of the PI3K/Akt pathway, which takes part in cell proliferation, migration, differentiation, and protein synthesis [43]. Studies have shown that the hypoxia-induced PAH model indicates that stimulating the PI3K/Akt pathway may promote abnormal proliferation of smooth muscle cells [44]. Furthermore, regulation of the PI3K/Akt pathway may influence the apoptosis of these cells. Thus, these and the proposed findings allude to the PI3K/Akt signaling pathway indirectly influencing the incidence of PAH.

The study found that macrophages, mast cells, and Th1 cells played a crucial role in the progression of PAH through immune cell infiltration. Research has acknowledged that macrophages play an essential role in the development of pulmonary vascular disease and may also contribute to its severity [45]. Mast cells are the first immune cells observed in pulmonary vascular lesions in individuals with PAH. Upon activation, mast cells release cysteine leukotriene C4 and endothelin, leading to pulmonary vascular remodeling and the onset of PAH [16]. Th1 cells contribute to the development of atherosclerosis. Th1 cells secrete cytokines, leading to inflammation in atherosclerotic lesions, and promoting the formation of foam cells [46]. The literature has not reported any relationship between Th1 cells and PAH. Conversely, studies suggest that immune-related functions such as APC-costimulation, checkpoint, para-inflammation, and T-cell-costimulation play a significant role in PAH development. A correlation between immune infiltration and the presence of inflammatory BM in PAH patients was found in this study. Further exploration should be conducted on the creation of miRNA regulatory networks. Additional investigations are required to ascertain the potential of integrated immune-infiltration-based immune cell analysis and ceRNA network construction for identifying diagnostic markers that can advance clinical management and treatment options for PAH. Simultaneously, researchers have focused on examining the genes responsible for the pathological progression of the condition and discovering targeted therapies that are effective. Ginsenoside Rh1 is a notable instance. Studies have demonstrated the positive effects of ginsenoside Rh1 on myocardial remodeling and pulmonary hemodynamics in PAH patients [47]. However, further *in vivo* and *in vitro* experiments are necessary to validate its efficacy and safety.

Nevertheless, our study has some limitations. First, there is a scarcity of research studying the mechanisms that underlie the correlation between BM and PAH. In addition, the current research sample is limited, which may introduce potential bias. Although six hub genes related to the development of PAH have been identified, further experiments are required to confirm their regulatory role in the pathogenesis of PAH.
